# Supplementary material for: Sorting at embryonic boundaries requires high heterotypic interfacial tension
Source: Nat Commun. 2017 Jul 31;8:157. doi: 10.1038/s41467-017-00146-x (PMC5537356; doi:10.1038/s41467-017-00146-x)
Supplement: Supplementary file 2 — Supplementary Software 1 [file 41467_2017_146_MOESM2_ESM.zip › PottsModel/SrcPottsModel/doc/model/package-use.html]

Uses of Package model


JavaScript is disabled on your browser.


Skip navigation links


- Overview
- Package
- Class
- Use
- Tree
- Deprecated
- Index
- Help

- Prev
- Next

- Frames
- No Frames

- All Classes

# Uses of Package model

- Packages that use model

  | Package | Description |
  | --- | --- |
  |  |  |
  | --- | --- |
  | engine |  |
  | gui |  |
  | model |  |
  | simulations |  |
- Classes in model used by engine

  Class and Description

  Cell

  Cell.CellType

  Constants

  Reads Constants from config file

  EnergyFunction.Energy

  KnowsConstants

  Lattice

  Lattice.PixelIterator

  Pixel
- Classes in model used by gui

  Class and Description

  Cell

  Constants

  Reads Constants from config file

  Coordinates

  IPositionManager.Position

  Lattice

  Pixel
- Classes in model used by model

  Class and Description

  CartesianCoordinates

  Cell

  Cell.CellType

  Cell.Energy

  Cell.State

  Enum class representing the different states of the cell that can be
  passed as a notification message to the different class observers.

  CellSet

  Constants

  Reads Constants from config file

  Constants.Model

  Constants.ParameterManager

  Manages energy information related to this simulation.

  Coordinates

  EnergyFunction

  EnergyFunction.Energy

  FiniteVector

  HalfLine

  IPositionManager

  IPositionManager.Position

  KnowsConstants

  Lattice

  Lattice.PixelIterator

  Lattice.State

  Lattice.Type

  Pixel

  Pixel.PixelType

  SquareCell

  Vector
- Classes in model used by simulations

  Class and Description

  Constants

  Reads Constants from config file

Skip navigation links


- Overview
- Package
- Class
- Use
- Tree
- Deprecated
- Index
- Help

- Prev
- Next

- Frames
- No Frames

- All Classes
